# Role of interoceptive accuracy in topographical changes in emotion-induced bodily sensations

**DOI:** 10.1371/journal.pone.0183211

**Published:** 2017-09-06

**Authors:** Won-Mo Jung, Yeonhee Ryu, Ye-Seul Lee, Christian Wallraven, Younbyoung Chae

**Affiliations:** 1 Acupuncture & Meridian Science Research Center, College of Korean Medicine, Kyung Hee University, Seoul, Republic of Korea; 2 Acupuncture, Moxibustion and Meridian Research Center, Division of Standard Research, Korea Institute of Oriental Medicine, Daejeon, Republic of Korea; 3 Department of Brain Cognitive Engineering, Korea University, Seoul, Republic of Korea; Ludwig-Maximilians-Universität München, GERMANY

## Abstract

The emotion-associated bodily sensation map is composed of a specific topographical distribution of bodily sensations to categorical emotions. The present study investigated whether or not interoceptive accuracy was associated with topographical changes in this map following emotion-induced bodily sensations. This study included 31 participants who observed short video clips containing emotional stimuli and then reported their sensations on the body map. Interoceptive accuracy was evaluated with a heartbeat detection task and the spatial patterns of bodily sensations to specific emotions, including anger, fear, disgust, happiness, sadness, and neutral, were visualized using Statistical Parametric Mapping (SPM) analyses. Distinct patterns of bodily sensations were identified for different emotional states. In addition, positive correlations were found between the magnitude of sensation in emotion-specific regions and interoceptive accuracy across individuals. A greater degree of interoceptive accuracy was associated with more specific topographical changes after emotional stimuli. These results suggest that the awareness of one’s internal bodily states might play a crucial role as a required messenger of sensory information during the affective process.

## Introduction

When encountered with an emotional stimulus, event, or context in daily life, humans experience associated emotions as well as corresponding physiological reactions within the body. In this context, an emotion is considered the perceived central representation of bodily responses to environmental stimuli [1]. Based on this idea, William James insisted a hypothesis that different emotional states should be characterized by differential bodily states. This hypothesis was challenged by Cannon-Bard theory [2], which articulated that visceral responses are too uniform and slow to be sources of distinct various emotions. Instead of emphasizing the visceral source of emotion, Cannon emphasized the role of central nervous system to generate emotional feelings. Nevertheless, a number of evidences [3–5] corroborate the importance of somatic feedback in emotional experience. Specific physiological reactions during an emotional state involve various physiological systems, including the cardiovascular, skeletomuscular, neuroendocrine, and autonomic nervous systems [3, 4]. Damasio’s somatic marker theory proposes that somatosensory feedback from a physiological reaction triggers mental experiences and represents the origin of emotional feelings [6, 7] and, accordingly, it has been shown that specific combinations of somatosensory and visceral afferents are fundamental for building up emotional feelings [8]. Importantly, Nummenmaa *et al*. [8] demonstrated that consciously accessible somatosensory feelings in certain emotional contexts are spatially distributed onto the body in a specific and consistent pattern. One of the possible hypotheses of the origin of emotion-specific bodily sensation is that the emotion-specific somatotopic bodily sensation may reflect the specific physiological state of each emotion. This approach to understanding emotions has also demonstrated that quantifiable univariate physiological signals, such as heart rate and skin conductance, can be informative sources for the emotional process and that there is a perceived spatial distribution of these signals on the body [5].

Interoception, which is the awareness of one’s internal bodily states, plays key roles in the emotional experience, the processing of emotional stimuli, and the activation of brain areas that monitor the internal visceral and emotional states of organisms [9, 10]. Interoceptive accuracy is commonly indexed with objective measures of the ability to detect one’s own heartbeat [11] and a greater ability to perceive these types of visceral responses is thought to reflect more intense subjective states as well as the enhanced integration of bodily signals into the emotional experience [12]. For example, individuals who can better detect their own heartbeats experience emotions with a heightened intensity [13]. Similarly, good heartbeat detectors show traits of emphasizing their emotional experiences compared to others, as measured by the degree of arousal focus [14]. Furthermore, imaging studies of the brain have provided support for the role of centrally integrated feedback from the whole body during emotional awareness as evidenced by activities in the anterior insula and anterior cingulate cortex during both interoception and emotion [10, 12]. In this context, individual differences in emotional awareness are significantly associated with variation in the capacity for interoceptive feelings [9]. Although body awareness in and of itself is a popular topic along with interoception, no studies have investigated whether interoceptive accuracy may influence the topographical maps of bodily sensation in different emotional states.

Thus, we investigated whether emotion category-specific bodily sensations exist, to determine whether somatotopic patterns of bodily sensation differ as the degree of interoceptive sensitivity varies. Based on empirical evidence showing that the bodily state is a key component of emotional responses [13–19], it was hypothesized that individuals with higher levels of interoceptive accuracy about their bodily state (as measured by a heartbeat-detection task) would be more likely to experience stronger sensations in emotion-specific bodily locations.

## Materials and methods

### Participants

This study included 31 healthy human volunteers (age: 24.1 ± 4.5 years; 15 females) who were recruited via advertisement. None of the participants had any history of neurological, psychiatric, or other major medical problems and no participants were using medications at the time of the study. In addition, participants were instructed not to drink alcohol or caffeine and take any medications while participating in the study. All participants provided written informed consent prior to the experiments and the Institutional Review Board at Korea University approved all study procedures. After providing the informed consent, participants were required to fill out the empathy quotient questionnaire. This questionnaire was developed by Baron-Cohen et al. [20], in order to measure the empathic characteristics of normal population. This questionnaire consisted of 40 empathy-related questions and 20 control questions, with a maximum achievable score of 80. Kim et al. [21] translated the questionnaire into Korean, and validated the translated version. We used the validated Korean version of empathy quotient scale.

### Emotional visual stimuli

Similar to the previous study [8], various types of emotional stimuli were used to minimize possible biases. We prepared two types of emotional stimuli, emotional graphics interchange format (GIF) images and dynamic facial expressions, which were used to elicit the following discrete emotional states: anger, fear, disgust, happiness, sadness, and neutral.

One type of stimuli, the emotional GIF images, was derived from an online platform (http://www.gif.gf/) which collects emotional impact ratings of their images from the online raters. The evaluation of the GIF images is based on about 1,000 votes per image. This platform provides a list of the ranking of the images for each discrete category of emotions. We downloaded top 40 GIF images for each emotion in the present study (anger, fear, disgust, happiness, sadness, and neutral). Among the 40 GIF images, ten relevant GIF images per each emotion were selected while excluding animated cartoon clips or popular movie clips. Ten selected images were divided into two groups of five, so that five images were shown in succession in one trial. Each GIF image was presented for 2 seconds and continuously repeated twice. Thus, the whole presentation of GIF images in a trial was 20 seconds long.

The other type of stimuli, dynamic facial expressions, was obtained from the Dynamic Facial Expressions Database of Korea University [22]. For this database, actors performed 62 expressions according to a method-acting protocol by imagining themselves in 1 of 62 corresponding everyday scenarios and then reacting accordingly. The dynamic facial expressions corresponding to the emotions in the present study were selected from stimuli that were successfully used to induce emotion in a previous functional magnetic resonance imaging (fMRI) experiment [23]. For the neutral state, facial expressions were selected from the “thinking and considering” scenario. Ten stimuli of each type were selected for each emotion, which were posed by five female actresses and five male actors. Like the GIF image, ten images were divided into two groups of five, so that five images were shown in succession in one trial. Each dynamic facial expression was presented for 4 seconds, thus the whole presentation of facial expressions in a trial was also 20 seconds long.

**Fig 1:**
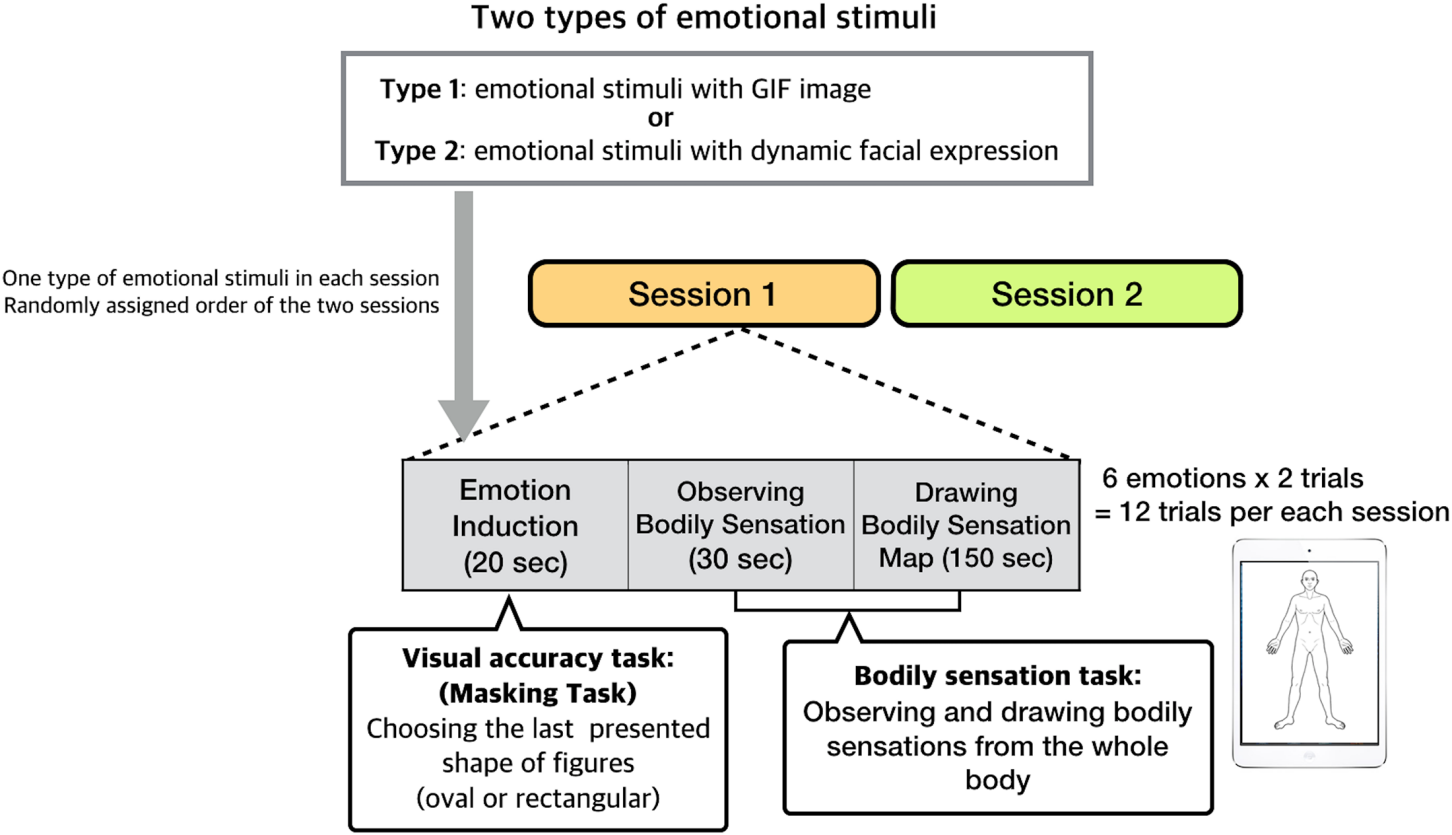
Experimental design and procedure. The entire experimental procedure was divided into two sessions of 12 trials each with one trial consisting of two consecutive tasks: the visual accuracy task and the bodily sensation task. During the visual accuracy task, participants were asked to observe short video clips that contained emotional stimuli and then report their internal sensations from the whole body to create the observed bodily sensations on a body map. Two types of emotional stimuli were used to elicit discrete emotional states, including anger, fear, disgust, happiness, sadness, and neutral: emotional GIF images and dynamic facial expressions.

The two types of emotional stimuli were separated into two sessions of 12 trials; two trials per each emotion (Fig 1). Since a record of one bodily sensation was obtained after each presentation of grouped emotional stimuli, four bodily sensations were collected for each emotion. Presentation of a group of images (20 sec) was separated by a 3 min interval, and the order was randomly assigned. During the interval, the bodily sensation task and the consecutive bodily sensation collection were performed.

### Experimental design and procedure

Participants performed two consecutive sessions that included two different tasks: a visual accuracy task and a bodily sensation task. After being exposed to emotional stimuli during these sessions, the participants recorded their bodily sensations in the form of spatial information or bodily sensation maps drawn using software. All participants were given the following instructions: “This experiment was intended to investigate whether or not bodily sensation tasks are different depending on different levels of visual accuracy tasks.” Bodily sensation maps were measured immediately after viewing the emotional stimuli during the visual accuracy tasks. To minimize any type of bias while drawing the bodily sensation map, due to possible conceptions on the relationship between the emotion and the body parts, we disguised the intention of our experimental task which was to induce emotional states.

During the visual accuracy task, the participants were asked to watch short video clips containing emotional stimuli that were disguised by the task. During the video clip, a random distracting figure was briefly shown (0.1 s) at the center of the emotional video clip. One or two distracting figures were presented for each trial. The figure was transparent and oval or rectangle shaped; only the outline was drawn in red. The width of the figure was 50% to the video clip. The oval or rectangle-shaped figure suddenly appeared once or twice during a video clip. The timing of the presentation was also randomly selected in a uniform probability model. The participants were asked to focus their attention on the random presentations of these figures. Then, at the end of each trial, they were asked to choose the shape of the last figure. Throughout the entire experiment, no emotion-related instructions was given, nor was the word “emotion” explicitly mentioned, which required the participants to focus on the given task. The difficulty level of the masking task was set high, since our aim was to make it difficult for the participants to realize the actual purpose of the experiment by focusing on the masking task. The group mean of the task accuracy was not significantly different from the chance level (*p* = 0.304), confirming that that the task was difficult for the participants. Also the task accuracy did not change as a function of emotion. One way ANOVA test was applied to test task accuracy varies between emotions. But, we could not find any significant difference of task accuracies between emotions (F _(5, 25)_ = 0.388, *p* = 0.856).

There were 12 emotion induction tasks (videos/visual accuracy trials) in each session (total 24). One bodily sensation task followed each emotion induction task consecutively; therefore, the bodily sensation tasks were carried out 24 times in total for two sessions. During the bodily sensation task, the participants were asked to identify bodily sensations from their whole body and to draw their observations on a body map; in this manner, they were asked to “interocept” (i.e., look into their body) for 30 s to define the sensations while their vision and audition were blocked with an eye patch and ear muffs. Participants were given the following instructions: “Close your eyes until you hear a beep sound at the end of the 30-second period, and carefully look into your bodily sensations and where your senses come from.” During the task, the participants were positioned in a comfortable arm chair reclined at 30 degrees with respect to the floor.

### Measurement of bodily sensation

Following the bodily interoception task of looking into bodily sensation, the participants were asked to report the locations of their bodily sensations. It was allowed not to draw on any part of the bodily sensation map when no sensation was felt. To accomplish this, a bodily sensation map-emotion (BSM-E) application, which presents a template of the human body as two-dimensional frontal images (1,536 × 2,048 pixels; http://cmslab.khu.ac.kr/downloads/bsm), was used on an iPad (Apple Inc.; Cupertino, CA, USA) [24, 25]. With this application, the user can change the color of the points on a continuous color map via successive strokes on a region with a touch pen (Wacom Inc. OR, USA).

### Heartbeat tracking task

After the two task sessions, the interoceptive accuracy of each participant was measured with the standard heartbeat-tracking task [26]. Participants were seated upright in a quiet room with their eyes closed and asked to silently count their heartbeats; the beginning and end of the counting phases were signaled by soft start and stop tones. This task was repeated three times within time windows of 15, 30, and 45 s that were presented in a randomized order and separated by a standard resting period of 30 s. Participants were given the following instructions: “Without speaking out loud, please track and count the heartbeat you feel in your body from the time you hear the starting signal until you hear the stop signal.” While counting their heartbeats, the participants were not allowed to take their pulse and they did not receive feedback regarding the length of the counting phases or the quality of the performance provided.

During the same period, the heartbeat signals were acquired by an electrocardiogram amplifier via electrocardiogram electrodes placed on the thorax (BioAmp ML132, AD Instruments; Bella Vista, Australia). For each time window, an accuracy score was calculated using the following equation: 1 –(|number of beats (actual)–number of beats (reported)|)/((number of beats (actual)+number of beats (reported))/2). Final accuracy scores were averaged over three trials and a maximum score of 1 indicated the veridical accuracy of heartbeat perception [26].

### Data analysis

A priori sample size was estimated prior to the experiment, which showed that 27 subjects would be required to detect a minimum ratio of the explained variance of 20% (R^2^ = 0.2) at an α level of 0.05 and 80% power. Statistical analyses of the bodily sensation data were performed using python libraries and Analysis of Functional Neuroimages (AFNI) software. To represent the spatial patterns of the sensations under each emotional state, the parametric maps of bodily sensations for emotional state were extracted from numerical matrices derived from the BSM-E application.

Individual datasets for each subject and for each trial were normalized within the range of 0–1. The normalized BSMs were subjected to three group-level analyses of statistical parametric maps: 1) emotion-specific bodily sensation map without distinguishing the types of stimuli (GIF image and dynamic facial expression), 2) emotion-specific bodily sensation map for each of two stimuli types, and 3) pairwise comparisons of bodily sensation maps between emotions. First, specific patterns of bodily sensation without distinguishing the types of stimuli (GIF image and dynamic facial expression) were investigated for each emotion (anger, fear, disgust, happiness, sadness, and neutral). For the statistical test of emotion-specific bodily sensation map, pixel-wise univariate t-test was applied for each emotion (3dttest++, AFNI, http://afni.nimh.nih.gov/afni) within a mask of the body template. In the all analyses of statistical parametric maps, family-wise error (FWE) correction was used to handle false positives due to multiple comparisons. To obtain a corrected type I error of *p* < 0.05 for the whole body of data, 10,000 Monte Carlo simulations were performed with the AFNI AlphaSim package, and a corresponding cluster size of at least 765 significant pixels with an individual FDR-corrected *p* < 0.05 was determined [27]. Second, bodily sensation maps were classified as the stimuli types and tested in the same manner except that additional paired *t*-test between stimuli types was applied for each emotion. Third, the discriminability of bodily sensation patterns for emotions was investigated by comparing bodily sensation maps by pairs using paired *t*-test. Because of ten pairwise matchings between emotions, a total of 10 comparisons were made. Before thresholding out the clusters by size, the number of pairwise matching was considered in the calculation of FDR value of individual pixel-wise thresholding. In the resulting statistical maps, the *t*-values for each pixel were transformed into Z-scores that reflected significant spatial information regarding bodily sensations. Similar to fMRI-activation maps, the color code was visualized according to the Z-score.

We also investigated the relationship between interoceptive accuracy and bodily sensations after exposure to emotional stimuli. First, emotion-specific regions of interest (ROIs) on the body map were defined based on the group analysis and then the correlation between the extracted sensation magnitude from the defined ROIs and interoceptive accuracy across individuals was determined. Because the heartbeat tracking task was performed only once during the entire experiment, one interoceptive accuracy value per individual was used for the correlation analysis. Emotion-specific ROIs were defined by selecting the peak points of significant clusters using a threshold of an FDR-corrected *p* < 0.05 and a cluster size of less than 765 for each emotion. Next, circular areas with a radius of 10 pixels from the peak points were defined as emotion-specific ROIs and a normalized bodily sensation magnitude within one trial report was extracted for each emotion-specific ROI. Finally, an exploratory correlation analysis between the averaged sensation magnitude across emotions except for the neutral condition from the ROIs and the individual interoceptive accuracies was performed. We provided the location of ROIs for each emotion in S1 Fig. To exclude possible confounding factors, some auxiliary correlation analyses were performed on 1) the relationship between the size of the drawn area and interoceptive accuracy, 2) the relationship between the performance of the visual accuracy task and the averaged sensation magnitude in ROIs, and 3) the relationship between the empathy scale and the averaged sensation magnitude in ROIs. All of these analyses were based on Pearson's correlation test on individual values of participants.

For additional information, body areas showing correlations between sensation magnitude and interoceptive accuracy as measured across individuals were visualized using the analysis of covariance (ANCOVA) function provided in the 3dttest++ program (AFNI, http://afni.nimh.nih.gov/afni). A threshold of |*r*| > 0.2 was applied to visualize the general broad-scale pattern of the correlation coefficients.

## Results

### Emotion-specific bodily sensation maps

**Fig 2:**
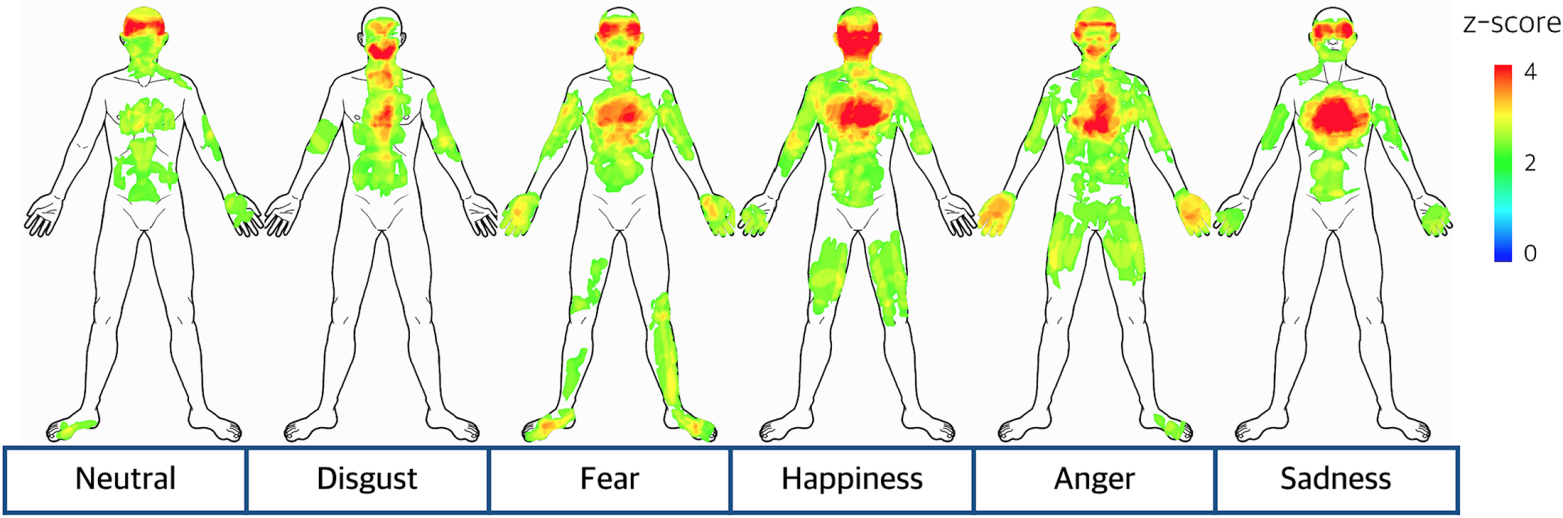
Emotion-specific bodily sensation map. Significant patterns of bodily sensation were identified for each emotion (anger, fear, disgust, happiness, sadness, and neutral). Statistical values were transformed into Z-scores that indicated the significance of a sensation at the group level and then mapped on a front-posed body template.

**Fig 3:**
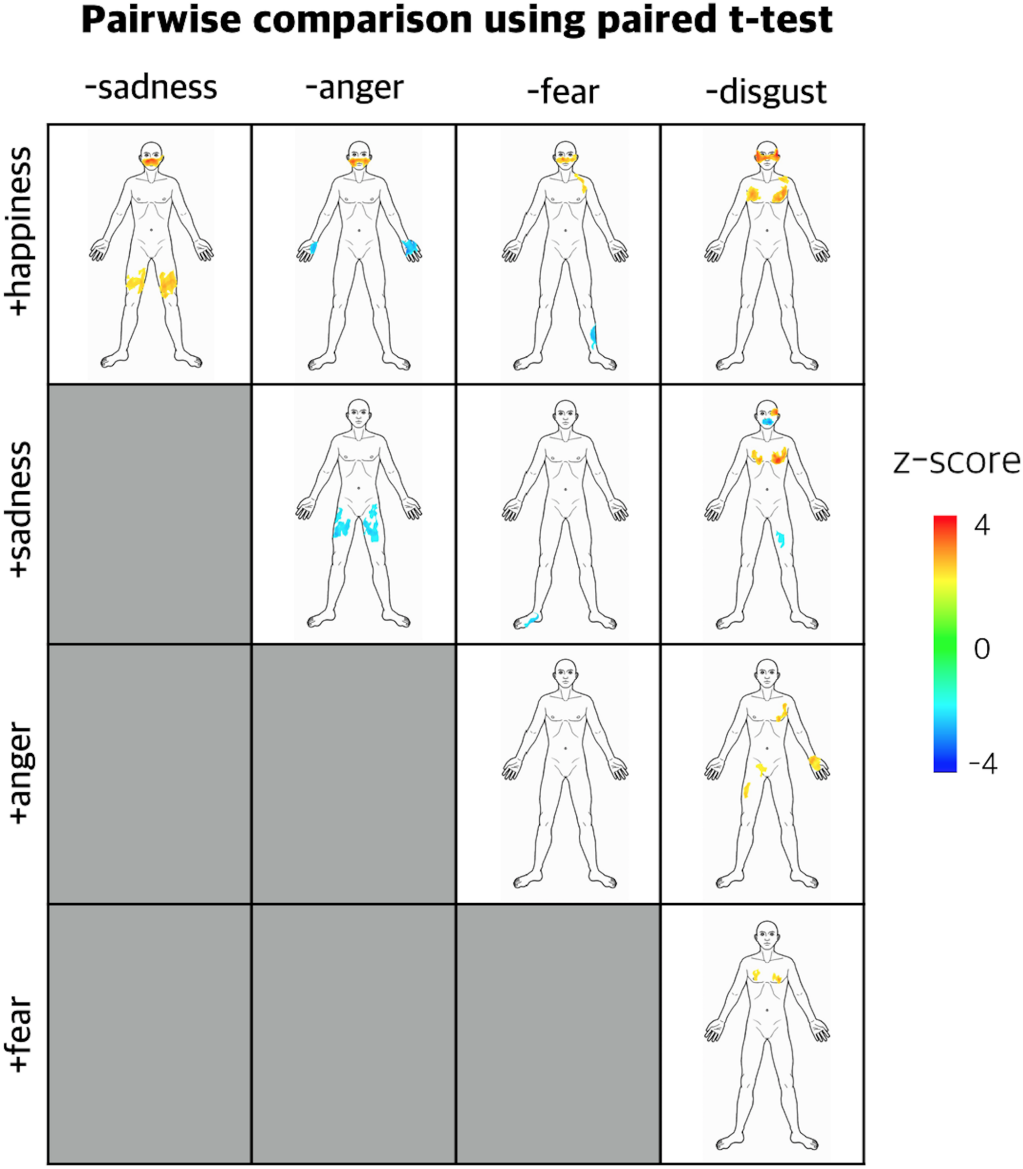
Statistical parametric maps comparing emotions by pairs using paired t-test. The paired statistical maps are displayed in a tabular layout. Rows in the table indicate emotions calculated as positive variables in the paired *t*-test. Columns in the table indicate emotions calculated as negative variables in the paired *t*-test. A total of 10 comparisons were made from the pairwise matching between emotions. Cluster corrected results at *p* < 0.05 level are presented. Statistical values were transformed into Z-scores, which indicated the significance of a sensation at the group level, and then mapped on a front-posed body template.

Significant bodily sensation patterns were visualized for each emotion assessed in the present study (anger, fear, disgust, happiness, sadness and neutral; Fig 2). Although all emotions exhibited a common pattern of bodily sensation in the chest area, specific spatial patterns of bodily sensation were also observed for each emotion. Both anger and fear showed highly reliable sensations in fists while only fear exhibited bodily sensations in the lower legs and foot. Disgust showed a specific bodily sensation pattern along the gastrointestinal tract. The bodily sensation maps of emotions were compared by pairs of emotions. The pairwise comparison of the bodily sensation patterns of emotions using paired *t*-test showed that bodily sensation patterns for emotions were discriminable in group level (Fig 3).

Furthermore, we used two different types of emotional stimuli (GIF image and dynamic facial expression) in this study. There was no significant difference between the two types of bodily sensation patterns when we compared them using paired *t*-test (at the cluster-corrected significance level of *p* < 0.05). Visualization of emotion-specific bodily sensation map by stimuli types was shown in S2 Fig.

### Correlations between emotion-specific bodily sensations and interoceptive accuracy

**Fig 4:**
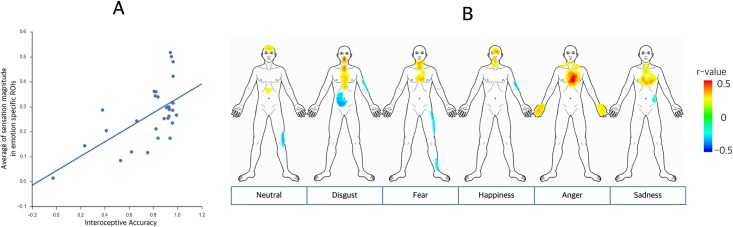
A) Correlations between emotion-specific bodily sensations and interoceptive accuracy. Significant positive correlations were found between the average sensation magnitude in emotion-specific ROIs and interoceptive accuracy as measured by the heartbeat tracking task across individuals (*r* = 0.367, *p* = 0.042). Individual participants were scattered on the two axes (x-axis: interoceptive accuracy and y-axis: average sensation magnitude in emotion-specific ROIs). B) Visualization of the covariate map to individual interoceptive accuracy. The regions showing positive correlations with interoceptive accuracy matched well with regions showing a significant relationship with each emotional state (anger, fear, disgust, happiness, sadness, and neutral). The general distributed pattern of correlation coefficients was visualized with a threshold of |*r*| > 0.2.

Positive correlations were observed between the average sensation magnitudes in the emotion-specific ROIs for all emotions and interoceptive accuracy as measured by the heartbeat-tracking task across individuals (*r* = 0.367, *p* = 0.042; Fig 4A). These findings suggest that individuals with more accurate interoception had stronger sensations in emotion-specific bodily locations.

In contrast, the visual accuracy task was not significantly correlated with sensation magnitude in the emotion-specific ROIs (*r* = -0.090, *p* = 0.628). This finding suggests that the participants’ concentration during the task did not have an influence on the stronger sensations in the emotion-specific bodily locations.

### Visualization of the covariate map to individual interoceptive accuracy

The regions showing positive correlations with interoceptive accuracy matched well with the regions exhibiting a significant relationship with each emotion (Fig 4B) and the distributed pattern of the regions with positive correlations was distinguishable across emotions. Hence, individuals with greater interoceptive accuracy had more specific body sensations towards the emotional stimuli.

## Discussion

The bodily sensation maps of individuals exhibited distinguishable patterns that were specifically associated with particular emotions. In addition, we found for the first time that individuals with greater interoceptive accuracy experienced a greater degree of bodily sensation in emotion-specific body regions following exposure to emotional stimuli. As the emotion specific body regions were selected from the highest local Z-scores in the significant clusters in the emotion-specific bodily sensation map, the positive correlation between the interoceptive accuracy and the average sensation magnitude in the regions provides evidence that the higher the interoceptive accuracy, the closer the sensation pattern to the universal sensation pattern of participants. As hypothesized, interoceptive sensitivity may influence emotion-induced bodily sensations. These findings provide novel insights that will contribute to the current understanding of the role that emotion-specific bodily sensation patterns play in the process of feeling emotions.

We found that individuals exhibited emotion-specific activation maps after exposure to emotional stimuli. This finding is consistent with a previous study that showed that lexical or visual information of different emotions can be represented in an embodied somatotopic format and that emotion-triggered bodily changes generate culturally universal categorical experiences of different emotions [8]. The previous study tested the discriminability of the different bodily maps with cross-validated classification performance using k-Nearest Neighbors algorithm. This study, on the other hand, provided statistical parametric maps comparing emotions by pairs using paired *t*-test (Fig 3). The result in our study is in line with the previous study by showing that bodily sensation patterns for emotions were discriminable in group level. Taken together, these findings suggest that there is a shared recognition of distinct bodily sensation patterns for specific emotions, without the influence of cultural conceptions.

In the previous study by Nummenmaa [8], it was obviously exposed to the participants that the aim of the experiment was to investigate the association between emotion and bodily sensation. Thus, there is a possibility that the conceptual association between emotion and bodily sensation has influenced the results. However, the previous study tried to minimize the possibility of conceptual association between emotion and bodily sensation by using various types of emotional stimuli, and validating results with participants from different cultural backgrounds. On the other hand, our study disguised the original purpose of our experiment in order to minimize the conceptual influence by putting the masking task (visual accuracy task) on the front. Thus, these results corroborate the evidence that the topographical patterns of bodily sensations may be an informative source of affective perception, even when the conceptual bias is minimized.

We also found that individuals with greater interoceptive accuracy exhibited more specific topographical changes in emotion-induced bodily sensations. Although we did not investigate the emotional impact of the subjects during the emotional stimulation in order to conceal our original experimental purpose, there are several evidences [10–13] supporting the association between the interoceptive awareness and the quality of one’s emotional experience. Notably, alexithymia, which is characterized by difficulties recognizing emotions, is associated with interoceptive impairments [9, 19, 28]. On the other hand, studies of interoception have shown that increasing the accuracy and reliability of bodily signals impairs the malleability of one’s body representation [29]. By extending the relationship between interoception and emotion, the concept of predictive coding has yielded novel insights into emotion. This extension of a predictive perspective from exteroceptive perception (e.g., vision, audition, and so forth) to interoceptive physiological signals (e.g., heartbeat, respiration, and so forth) allows for emotion to be viewed as a subjective feeling state that is generated by the active top-down inference of the causes of interoceptive afferent signals [30]. These converging lines of evidence support the argument that emotional feelings may be based on the active inference of interoceptive afferent signals from the body. Taken together, the notion that emotion-specific topological patterns of bodily sensations are driven by interoceptive sensitivity may play a crucial role in differentiating and refining the presentation of internal feelings which, in turn, may influence emotional experiences.

The present study had several limitations that should be noted. First, to exclude the influence of prior sociocultural knowledge, we disguised original experimental purpose by employing the masking task or the visual accuracy task, which covers the relationship between emotional stimuli and bodily sensations. However, because the emotional stimuli were not implicitly exposed to the participants, it was not possible to fully exclude the influence of cultural preconceptions on the emotion-specific bodily sensation map. Second, we did not measure subjective emotional experience because the original purpose, inducing emotional state, was masked by the visual accuracy task. This prevented confirmation of the finding that individuals with higher interoceptive accuracy experienced stronger emotional feelings. Nonetheless, our results provide strong evidence that individuals with greater interoceptive accuracy exhibited more specific topographical changes in emotion-induced bodily sensations. Third, we observed somatotopic patterns following exposure to emotional stimuli that were similar to those reported by a previous study [8]. However, we also found distinct patterns of bodily sensations in the forehead following the neutral stimuli. This discrepancy in spatial patterns may have been due to the use of dynamic facial expressions from the “thinking and considering” category for the neutral states in the present study. Fourth, there might be underlying factors in the relationship between individual accuracy and specific topographical changes in bodily sensation for emotion. We found no significant correlation between the empathy scale and specific topographical changes in the bodily sensation for emotion (*r* = -0.031, *p* = 0.866). According to this result, empathy was not an underlying confounding factor in the relationship between individual accuracy and specific topographical changes in bodily sensation for emotion. However, we could not rule out other possible underlying factors, such as alexithymia.

In summary, the present study provides additional support of the specific spatial distributions of bodily sensations according to different emotions following exposure to emotional stimuli. These findings highlight the positive relationship between interoceptive sensitivity and patterns of bodily sensations induced by emotion and support the idea that physiological feedback from the body may play a crucial role in the emotional experience. Given the importance of interoception in emotion-induced bodily sensations, interoceptive observations may act as a visible presentation of affective perception and its link with physiological states. Thus, the topographical pattern of bodily sensations may be a fundamental response and sign of emotional coherence and the emotional experience.

## Supporting information

S1 Fig The location of ROIs for each emotion. Colored area of each body template indicates the location of the ROI in each emotion. The bodily sensation magnitude was estimated through the average of the extracted values from the ROIs. Emotion-specific ROIs were defined as the circular area with a radius of 10 pixels from the peak points of significant bodily sensation clusters. The size of the ROI was defined to be small to control for the possible confounding due to the size of the drawn area. To further exclude the possibility of a relationship between the size of the drawn area and the interoceptive accuracy, the size of the sensory area was calculated by the number of drawn pixels, and the Pearson’s correlation test was applied between the size of drawn area and the individual interoceptive accuracy. The analysis confirmed that there was no significant correlation between size of drawn area of the body map and interoceptive accuracy (*r* = 0.191, *p* = 0.301).
(DOCX)

S2 Fig Visualization of emotion-specific bodily sensation map by stimuli types (GIF images and dynamic facial expressions). Cluster corrected results at *p* < 0.05 level are presented as in the main results of Fig 2. Statistical values were transformed into Z-scores, which indicated the significance of a sensation at the group level, and then mapped on a front-posed body template.
(DOC)

## References

[1] James W. What is an emotion? Mind. 1884;9:188–205.

[2] Cannon WB. "Homeostasis" The wisdom of the body. Norton, Newyork 1932.

[3] Kreibig SD. Autonomic nervous system activity in emotion: a review. Biological psychology. 2010;84(3):394–421. doi: 10.1016/j.biopsycho.2010.03.010 . 20371374 10.1016/j.biopsycho.2010.03.010

[4] Levenson RW. Blood, sweat, and fears: the autonomic architecture of emotion. Annals of the New York Academy of Sciences. 2003;1000:348–66. . 14766648 10.1196/annals.1280.016

[5] Mauss IB, Levenson RW, McCarter L, Wilhelm FH, Gross JJ. The tie that binds? Coherence among emotion experience, behavior, and physiology. Emotion. 2005;5(2):175–90. doi: 10.1037/1528-3542.5.2.175 . 15982083 10.1037/1528-3542.5.2.175

[6] Bechara A, Damasio AR, Damasio H, Anderson SW. Insensitivity to future consequences following damage to human prefrontal cortex. Cognition. 1994;50(1–3):7–15. . 8039375 10.1016/0010-0277(94)90018-3

[7] Damasio A, Carvalho GB. The nature of feelings: evolutionary and neurobiological origins. Nature reviews Neuroscience. 2013;14(2):143–52. doi: 10.1038/nrn3403 . 23329161 10.1038/nrn3403

[8] Nummenmaa L, Glerean E, Hari R, Hietanen JK. Bodily maps of emotions. Proceedings of the National Academy of Sciences of the United States of America. 2014;111(2):646–51. doi: 10.1073/pnas.1321664111 ; 24379370 10.1073/pnas.1321664111 PMC3896150

[9] Herbert BM, Herbert C, Pollatos O. On the relationship between interoceptive awareness and alexithymia: is interoceptive awareness related to emotional awareness? Journal of personality. 2011;79(5):1149–75. doi: 10.1111/j.1467-6494.2011.00717.x . 21241306 10.1111/j.1467-6494.2011.00717.x

[10] Zaki J, Davis JI, Ochsner KN. Overlapping activity in anterior insula during interoception and emotional experience. NeuroImage. 2012;62(1):493–9. doi: 10.1016/j.neuroimage.2012.05.012 . 22587900 10.1016/j.neuroimage.2012.05.012 PMC6558972

[11] Wiens S. Interoception in emotional experience. Current opinion in neurology. 2005;18(4):442–7. . 16003122 10.1097/01.wco.0000168079.92106.99

[12] Critchley HD, Wiens S, Rotshtein P, Ohman A, Dolan RJ. Neural systems supporting interoceptive awareness. Nature neuroscience. 2004;7(2):189–95. doi: 10.1038/nn1176 . 14730305 10.1038/nn1176

[13] Wiens S, Mezzacappa ES, Katkin ES. Heartbeat detection and the experience of emotions. Cognition and Emotion. 2000;14(3):417–27.

[14] Barrett LF, Quigley KS, Bliss-Moreau E, Aronson KR. Interoceptive sensitivity and self-reports of emotional experience. Journal of personality and social psychology. 2004;87(5):684–97. doi: 10.1037/0022-3514.87.5.684 ; 15535779 10.1037/0022-3514.87.5.684 PMC1224728

[15] Craig AD. How do you feel? Interoception: the sense of the physiological condition of the body. Nature reviews Neuroscience. 2002;3(8):655–66. doi: 10.1038/nrn894 . 12154366 10.1038/nrn894

[16] Gu X, Hof PR, Friston KJ, Fan J. Anterior insular cortex and emotional awareness. The Journal of comparative neurology. 2013;521(15):3371–88. doi: 10.1002/cne.23368 ; 23749500 10.1002/cne.23368 PMC3999437

[17] Hogeveen J, Bird G, Chau A, Krueger F, Grafman J. Acquired alexithymia following damage to the anterior insula. Neuropsychologia. 2016;82:142–8. doi: 10.1016/j.neuropsychologia.2016.01.021 ; 26801227 10.1016/j.neuropsychologia.2016.01.021 PMC4752907

[18] Jones CL, Ward J, Critchley HD. The neuropsychological impact of insular cortex lesions. Journal of neurology, neurosurgery, and psychiatry. 2010;81(6):611–8. doi: 10.1136/jnnp.2009.193672 . 20522870 10.1136/jnnp.2009.193672

[19] Shah P, Hall R, Catmur C, Bird G. Alexithymia, not autism, is associated with impaired interoception. Cortex; a journal devoted to the study of the nervous system and behavior. 2016;81:215–20. doi: 10.1016/j.cortex.2016.03.021 ; 27253723 10.1016/j.cortex.2016.03.021 PMC4962768

[20] Baron-Cohen S, Wheelwright S. The empathy quotient: an investigation of adults with Asperger syndrome or high functioning autism, and normal sex differences. J Autism Dev Disord. 2004;34(2):163–75. . 15162935 10.1023/b:jadd.0000022607.19833.00

[21] Kim J, Lee SJ. Reliability and validity of the korean version of the empathy quotient scale. Psychiatry Investig. 2010;7(1):24–30. doi: 10.4306/pi.2010.7.1.24 ; 20396429 10.4306/pi.2010.7.1.24 PMC2848769

[22] Lee H, Shin A, Kim B, Wallraven C. The KU facial expression database: a validated database of emotional and conversational expressions. i-Percecption. 2012;3(694):2–35. 10.1371/journal.pone.0032321 PMC3305299 22438875

[23] Kim J, Schultz J, Rohe T, Wallraven C, Lee SW, Bulthoff HH. Abstract representations of associated emotions in the human brain. The Journal of neuroscience: the official journal of the Society for Neuroscience. 2015;35(14):5655–63. doi: 10.1523/JNEUROSCI.4059-14.2015 . 25855179 10.1523/JNEUROSCI.4059-14.2015 PMC6605320

[24] Jung WM, Lee SH, Lee YS, Chae Y. Exploring spatial patterns of acupoint indications from clinical data: A STROBE-compliant article. Medicine (Baltimore). 2017;96(17):e6768 doi: 10.1097/MD.0000000000006768 . 28445309 10.1097/MD.0000000000006768 PMC5413274

[25] Jung WM, Shim W, Lee T, Park HJ, Ryu Y, Beissner F, et al More than DeQi: Spatial Patterns of Acupuncture-Induced Bodily Sensations. Front Neurosci. 2016;10:462 doi: 10.3389/fnins.2016.00462 ; 27807402 10.3389/fnins.2016.00462 PMC5069343

[26] Hart N, McGowan J, Minati L, Critchley HD. Emotional regulation and bodily sensation: interoceptive awareness is intact in borderline personality disorder. Journal of personality disorders. 2013;27(4):506–18. doi: 10.1521/pedi_2012_26_049 . 22928847 10.1521/pedi_2012_26_049

[27] Ledberg A, Akerman S, Roland PE. Estimation of the probabilities of 3D clusters in functional brain images. NeuroImage. 1998;8(2):113–28. doi: 10.1006/nimg.1998.0336 . 9740755 10.1006/nimg.1998.0336

[28] Ernst J, Boker H, Hattenschwiler J, Schupbach D, Northoff G, Seifritz E, et al The association of interoceptive awareness and alexithymia with neurotransmitter concentrations in insula and anterior cingulate. Social cognitive and affective neuroscience. 2014;9(6):857–63. doi: 10.1093/scan/nst058 ; 23596189 10.1093/scan/nst058 PMC4040102

[29] Tsakiris M, Tajadura-Jimenez A, Costantini M. Just a heartbeat away from one's body: interoceptive sensitivity predicts malleability of body-representations. Proceedings Biological sciences / The Royal Society. 2011;278(1717):2470–6. doi: 10.1098/rspb.2010.2547 ; 21208964 10.1098/rspb.2010.2547 PMC3125630

[30] Seth AK. Interoceptive inference, emotion, and the embodied self. Trends in cognitive sciences. 2013;17(11):565–73. doi: 10.1016/j.tics.2013.09.007 . 24126130 10.1016/j.tics.2013.09.007

