# Supplementary material for: Role of interoceptive accuracy in topographical changes in emotion-induced bodily sensations
Source: PLoS One. 2017 Sep 6;12(9):e0183211. doi: 10.1371/journal.pone.0183211 (PMC5587262; doi:10.1371/journal.pone.0183211)
Supplement: S1 Fig — Colored area of each body template indicates the location of the ROI in each emotion. The bodily sensation magnitude was estimated through the average of the extracted values from the ROIs. Emotion-specific ROIs were defined as the circular area with a radius of 10 pixels from the peak points of significant bodily sensation clusters. The size of the ROI was defined to be small to control for the possible confounding due to the size of the drawn area. To further exclude the possibility of a relationship between the size of the drawn area and the interoceptive accuracy, the size of the sensory area was calculated by the number of drawn pixels, and the Pearson’s correlation test was applied between the size of drawn area and the individual interoceptive accuracy. The analysis confirmed that there was no significant correlation between size of drawn area of the body map and interoceptive accuracy (r = 0.191, p = 0.301). (DOCX) [file pone.0183211.s001.docx]

**
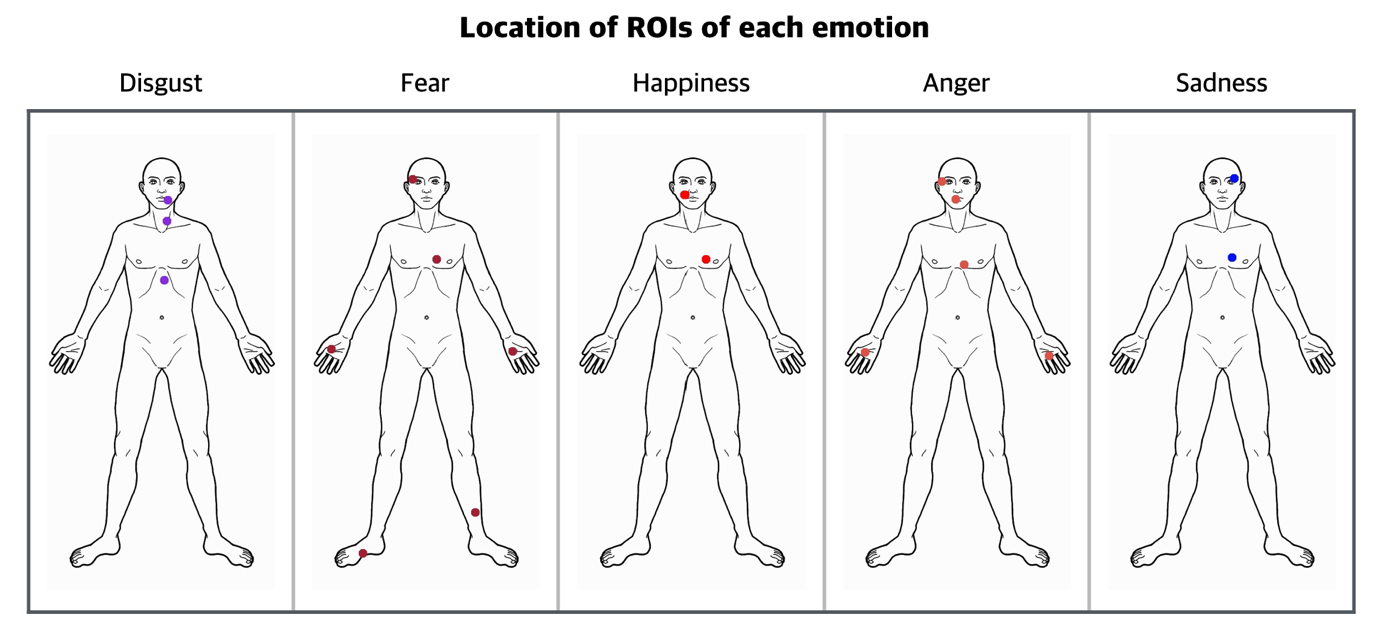
**

**S1 Fig.**

The location of ROIs for each emotion. Colored area of each body template indicates the location of the ROI in each emotion. The bodily sensation magnitude was estimated through the average of the extracted values from the ROIs. Emotion-specific ROIs were defined as the circular area with a radius of 10 pixels from the peak points of significant bodily sensation clusters. The size of the ROI was defined to be small to control for the possible confounding due to the size of the drawn area. To further exclude the possibility of a relationship between the size of the drawn area and the interoceptive accuracy, the size of the sensory area was calculated by the number of drawn pixels, and the Pearson’s correlation test was applied between the size of drawn area and the individual interoceptive accuracy. The analysis confirmed that there was no significant correlation between size of drawn area of the body map and interoceptive accuracy (*r* = 0.191, *p* = 0.301).
