# Supplementary material for: Role of interoceptive accuracy in topographical changes in emotion-induced bodily sensations
Source: PLoS One. 2017 Sep 6;12(9):e0183211. doi: 10.1371/journal.pone.0183211 (PMC5587262; doi:10.1371/journal.pone.0183211)
Supplement: S2 Fig — Cluster corrected results at p < 0.05 level are presented as in the main results of Fig 2. Statistical values were transformed into Z-scores, which indicated the significance of a sensation at the group level, and then mapped on a front-posed body template. (DOC) [file pone.0183211.s002.doc]

**
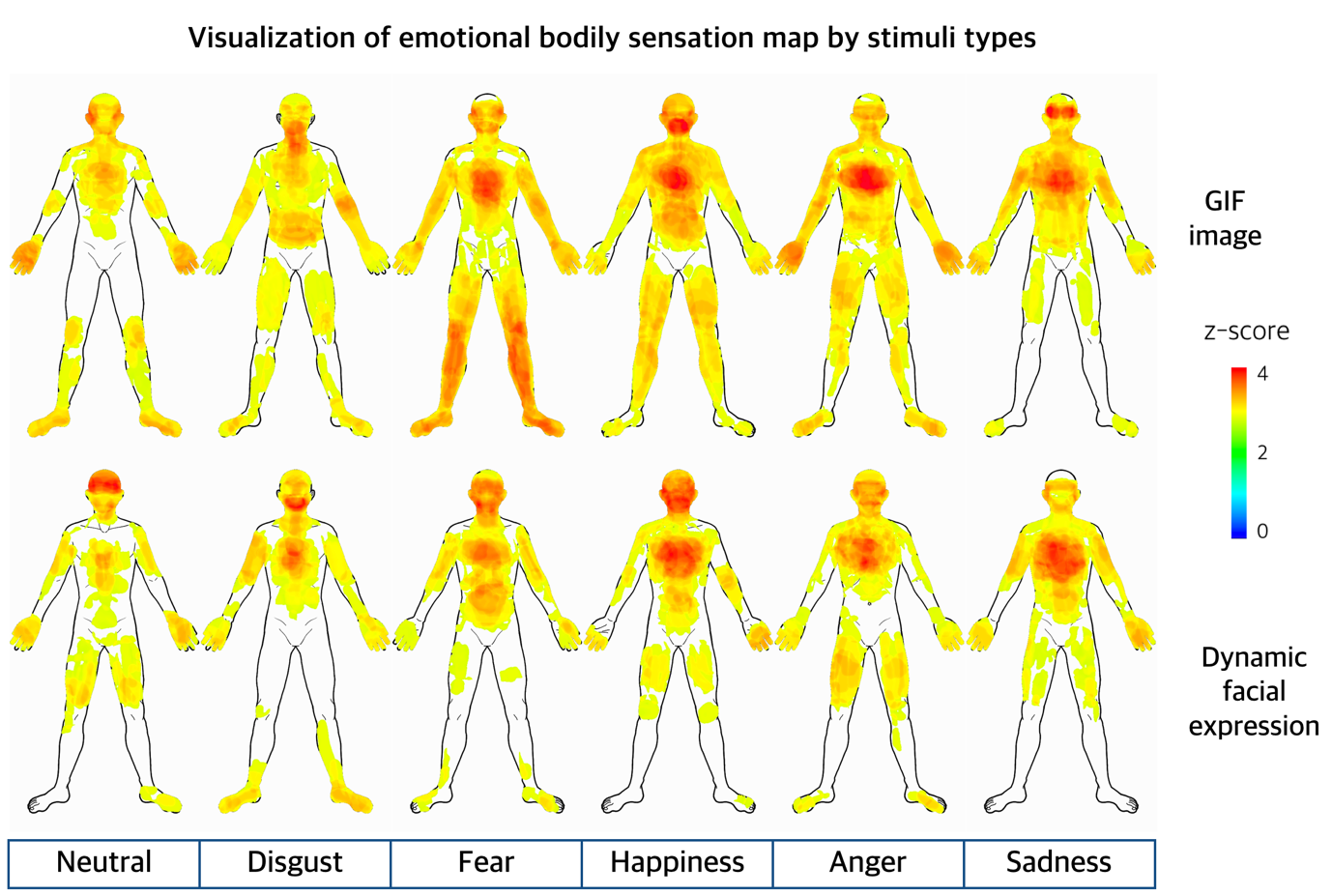
**

**S2 Fig.**

Visualization of emotion-specific bodily sensation map by stimuli types (GIF images and dynamic facial expressions). Cluster corrected results at *p* < 0.05 level are presented as in the main results of Figure 2. Statistical values were transformed into Z-scores, which indicated the significance of a sensation at the group level, and then mapped on a front-posed body template.
